# Pangenomic analysis identifies correlations between *Akkermansia* species and subspecies and human health outcomes

**DOI:** 10.20517/mrr.2024.09

**Published:** 2024-06-11

**Authors:** Katherine D. Mueller, M. Emilia Panzetta, Lauren Davey, Jessica R. McCann, John F. Rawls, Gilberto E. Flores, Raphael H. Valdivia

**Affiliations:** ^1^Department of Integrative Immunobiology, Duke University, Durham, NC 27710, USA.; ^2^Duke Microbiome Center, Duke University School of Medicine, Durham, NC 27710, USA.; ^3^Department of Biochemistry and Microbiology, University of Victoria, Victoria V8P 5C2, British Columbia, Canada.; ^4^Department of Molecular Genetics and Microbiology, Duke University, Durham, NC 27710, USA.; ^5^Department of Biology, California State University, Northridge, CA 91330, USA.

**Keywords:** *Akkermansia*, phylogeny, microbiome, IBD, obesity, immunotherapy

## Abstract

**Aim:** *Akkermansia* are common members of the human gastrointestinal microbiota. The prevalence of these mucophilic bacteria, especially *Akkermansia muciniphila* (*A. muciniphila*), correlates with immunological and metabolic health. The genus *Akkermansia* in humans includes species with significantly larger genomes than *A. muciniphila*, leading us to postulate that this added genetic content may influence how they impact human metabolic and immunological health.

**Methods:** We conducted a pangenomic analysis of 234 *Akkermansia* complete or near-complete genomes. We also used high-resolution species and subspecies assignments to reanalyze publicly available metagenomic datasets to determine if there are relationships between *Akkermansia* species and *A. muciniphila* clades with various disease outcomes.

**Results:** Analysis of genome-wide average nucleotide identity, 16S rRNA gene identity, conservation of core *Akkermansia* genes, and analysis of the fatty acid composition of representative isolates support the partitioning of the genus *Akkermansia* into several species. In addition, *A. muciniphila sensu stricto*, the most prevalent *Akkermansia* species in humans, should be subdivided into two subspecies. For a pediatric cohort, we observed species-specific correlations between *Akkermansia* abundance with baseline obesity or after various interventions. For inflammatory bowel disease cohorts, we identified a decreased abundance of *Akkermansia* in patients with ulcerative colitis or Crohn’s disease, which was species and subspecies-dependent. In patients undergoing immune checkpoint inhibitor therapies for non-small cell lung carcinoma, we observed a significant association between one *A. muciniphila* subspecies and survival outcomes.

**Conclusion:** Our findings suggest that the prevalence of specific *Akkermansia* species and/or subspecies can be crucial in evaluating their association with human health, particularly in different disease contexts, and is an important consideration for their use as probiotics.

## INTRODUCTION

*Akkermansia muciniphila* (*A. muciniphila*) is a Gram-negative, mucin-degrading bacterium that is prevalent in the mammalian gastrointestinal tract^[1,2]^. The relative abundance of *A. muciniphila* is associated with improved human health in a variety of contexts, such as obesity, metabolic disorders, responsiveness to cancer therapy, seizures, neuroinflammation, inflammatory bowel disease, and healthy aging^[3-12]^. Indeed, these associations with positive health outcomes have generated interest in developing *A. muciniphila* into a new class of probiotic for the treatment and prevention of multiple metabolic and immunological disorders^[13-16]^.

Most studies on the biology of *A. muciniphila* have focused on the original Muc^T^ (BAA-835) strain, which was isolated from human stool in the Netherlands^[1,2,17,18]^. When additional *A. muciniphila* isolates became available, Guo *et al*. first proposed three genetically distinct sub-species-level phylogroups, AmI, AmII, and AmIII^[19]^. As more isolates and genome sequences became available, additional phylogroups were proposed, including AmIV and AmV^[20,21]^. In addition, the *A. muciniphila* AmI phylogroup could be further subdivided into two sub-phylogroups, AmIa and AmIb^[20]^. Whole-genome analyses revealed potential phylogroup-specific metabolic differences. These were confirmed through the characterization of representative isolates demonstrating variations in growth rates in mucin medium, aerotolerance, ability to metabolize human milk oligosaccharides, vitamin B_12_ production, and activation of toll-like receptors (TLR)^[20-23]^. Based on the comparison of additional genomes from newly isolated human strains and near complete metagenome-assembled genomes (MAGs), there is an emerging consensus that genomes that were originally referred to as *A. muciniphila* should be sub-divided into several distinct species based on whole-genome nucleotide identity or conserved gene alignment^[24-28]^. Not all sub-species terminology used has been consistent with the original description of *Akkermansia* phylogroups ^[19]^, and the overlap in genomes used between studies has been limited. Recently, it was proposed that the AmIV phylogroup met the criteria of a new species of *Akkermansia* and was renamed *Akkermansia biwaensis* (*A. biwaensis*)^[29]^. A similar proposal was made for the AmII phylogroup to be renamed *Akkermansia massiliensis* (*A. massiliensis*)^[30]^. For consistency, we will refer to the AmII phylogroup as *A. massiliensis* and the AmIV phylogroup as *A. biwaensis.* Whether this assignment of new *Akkermansia* species or *A. muciniphila* phylogroups leads to different health outcomes remains an open question, especially since only one *Akkermansia* species at a time predominates within a single human host^[20]^. Of note, most reports exploring the role of *A. muciniphila* in human trials or preclinical murine models have focused on the AmIa strain Muc^T^^[15,31]^.

Given *in vitro* phenotypic differences between phylogroups that could be linked to interactions with its host, such as TLR stimulation and adherence to epithelial cells^[20]^, we postulate that which *Akkermansia* species is present in the GI tract may influence the ultimate impact on its human host. Indeed, one study found that *A. muciniphila* could be distinguished from *Akkermansia* candidate species based on marker genes from metagenome-assembled genomes^[25]^. Based on these markers, the authors suggested that the association observed between *A. muciniphila* and body mass index (BMI) did not apply to the additional candidate species. However, current analysis tools and databases^[32,33]^ do not, by default, distinguish between these *Akkermansia* phylogroups. In this study, we combined all the previously, but independently, described genomes from isolated strains into a single, large pangenome. Our pangenomic analysis defined a novel clade of *Akkermansia* and supported a species re-assignment for phylogroups AmII, IV, and V, which we corroborate with phenotypic characterizations of representative strains for each species and subspecies. Using these new classifications, we developed methods to identify *Akkermansia* species and subspecies from 16S rRNA and metagenomic sequences of stool samples. Finally, we provide three case studies to further support the premise that the relationship between the relative abundance of *Akkermansia* and health outcomes can correlate with specific *Akkermansia* phylogroups.

## METHODS

### Isolation of *Akkermansia* from fecal samples

Bacterial isolation and growth were performed in a Coy Laboratory anaerobic chamber under 5% hydrogen, 5% carbon dioxide, and 90% nitrogen. Eight *Akkermansia* strains were isolated from the stool of renal cell carcinoma (RCC) patients at Duke University Hospital (IRB protocol Pro00076768). Isolation of strains was performed as described previously^[20]^. Four *Akkermansia* strains were isolated from the stool of patients with amyotrophic lateral sclerosis (people with ALS, PALS) at Duke University Hospital (IRB protocol Pro00108282). To isolate new *Akkermansia* strains, approximately 100 mg of frozen stool was used to inoculate 1 mL of mucin medium as previously described^[1,20]^, and supplemented with cysteine (0.5 mM), vancomycin (6 μg/mL), gentamicin (10 μg/mL), and kanamycin (12 μg/mL) and incubated at 37 °C for 48 h. Three sequential passages at 1:10 dilutions were performed, and a sample was streaked on 1% agar mucin medium plates, supplemented with cysteine (0.5 mM), to facilitate the isolation of single colonies. After 7 days at 37 °C, individual colonies were picked and cultured in synthetic media^[5,20]^ supplemented with cysteine (0.5 mM).

### Genome sequencing and annotation

Sequencing and annotation of genomes labeled as “RCC” were performed as described previously^[20]^. For “PALS” genomes, genomic DNA extractions were carried out using the DNeasy Blood & Tissue kit (Qiagen catalog 69504) following the manufacturer’s protocol. DNA concentrations were determined using the Qubit double-stranded DNA (dsDNA) broad-range kit (Thermo Scientific). Library preparation and sequencing were performed by SeqCenter (https://www.seqcenter.com/). The Oxford Nanopore Technologies (ONT) Ligation Sequencing Kit (SQK-NBD114.24) with NEBNext® Companion Module (E7180L) was used to prepare DNA sequencing libraries, adhering to the manufacturer’s specifications. Genome sequencing was carried out on Nanopore R10.4.1 flow cells using the MinION Mk1B device. The Oxford Nanopore data processing toolkit, Guppy (v6.3.8), was used for base calling and demultiplexing. The super accurate model was used for base calling, as it offers the highest raw read accuracy^[34]^. The resulting fastq files were assembled into a single contig using Flye^[35]^. In addition, we used an Illumina DNA Prep kit and IDT 10bp UDI indices to prepare additional libraries, which were then sequenced on an Illumina NextSeq 2000, generating 2 × 151 bp reads. The Illumina software app bcl-convert (v3.9.3) is provided to convert files produced by Illumina systems into FASTQ format. We used this software to perform demultiplexing, quality control, and adapter trimming of reads^[36]^. To refine the Flye assembled genome, we used Pilon in combination with the Illumina reads for autocorrection^[37]^. The assembly underwent annotation and quality evaluation using the PATRIC genome annotation service^[38]^.

### Additional data access

Additional *Akkermansia* genomes (*n* = 221), including that of Muc^T^ and excluding MAGs, were retrieved from NCBI. A summary of each isolate can be found in Supplementary Table 1. The 16S rRNA-based microbial community profiling results in the form of a phyloseq object^[39]^ and raw metagenomic reads for validation of the proposed phylogroup prediction methods were obtained from the Pediatric Obesity Microbiome and Metabolism Study (POMMS)^[40]^. Metagenomic sequencing samples for the inflammatory bowel disease (IBD) and non-small cell lung cancer (NSCLC) datasets were obtained from the Sequence Read Archive using Bioproject accessions PRJNA398089, PRJNA400072, PRJEB42151, PRJEB42155, PRJNA751792 and PRJNA782662^[41-44]^.

### Pangenomic analysis

A comparison of 234 *A. muciniphila* genomes was performed using the pangenomic workflow in Anvi’o version 7.1^[45]^. Briefly, this workflow included construction of a pangenome from genome fasta files^[46,47]^, calculation of average nucleotide identity of assigned phylogroup^[48]^, core gene cluster identification and single-copy core protein sequence alignment^[49]^, estimation of functional enrichment^[50]^, and extraction of full-length 16S rRNA sequences^[51]^. An in-depth description of these methods and the parameters used may be found at https://gitlab.oit.duke.edu/valdivia-lab/akkermansia-species-and-phylogroups.

### Phenotypic characterization

Fatty acid methyl ester profiling of select strains was performed by EMSL Analytical, Inc. The API 20A system (bioMérieux catalog 20300) was used to measure 21 different biochemical phenotypes, including indole formation, urease and catalase activity, gelatin and esculin hydrolysis, and 16 acidification reactions. These tests were performed according to the manufacturer’s protocol under anaerobic conditions for 48 h.

### Analysis of community profiling data

The 16S rRNA-based microbial community profiling of the POMMS cohort was obtained as a phyloseq object^[40]^, which included the prevalence of identified amplicon sequence variants (ASVs). Identification of *Akkermansia* species and phylogroups from the data was performed using Phyloseq (v1.32.0)^[39]^ in R (v4.0.4)^[52]^. Raw metagenomic sequencing data were processed using the bioBakery suite^[53,54]^, StrainR^[55]^, and strain-level metagenomic estimation of growth rate (SMEG)^[56]^. Both the 16S rRNA and metagenomics analyses are detailed with examples at https://gitlab.oit.duke.edu/valdivia-lab/akkermansia-species-and-phylogroups. In all cases, POMMS samples with *Akkermansia* of known phylogroup were used as controls.

### Statistics and visualization

Figures were finalized using Inkscape^[57]^. Metabolic pathway completeness was visualized using the package pheatmap (v1.0.12) in R. Phylogenetic trees were visualized using the Interactive Tree of Life^[58]^. GraphPad Prism 10 (v10.1.0)^[59]^ was used to generate all remaining graphs, calculate the average ANI and 16S rRNA sequence identity between phylogroups, and perform statistical analyses. Principal Component Analysis of FAME results used the standardized method in GraphPad Prism. Mann-Whitney *U* tests were used to compare relative abundances in datasets containing two disease groups, Kruskal-Wallis H tests were used to compare relative abundances in datasets containing more than two disease groups, and Cox regression was used for survival analyses.

## RESULTS

### Genomic analysis of multiple *Akkermansia* isolates supports the establishment of new species and *A. muciniphila* subspecies

To perform our analysis, we retrieved 221 publicly available genome sequences originally identified as *A. muciniphila*, including Muc^T^, from NCBI. In addition to these, we added 13 new *Akkermansia* strains that we isolated from human and animal fecal samples. The final collection included 207 genomes from human *Akkermansia* isolates, 87 of which had been previously phylotyped as AmIa, AmIb, AmII, AmIII, AmIV, and AmV^[19-22,60]^, and 26 genomes derived from non-human sources.

**Figure 1:**
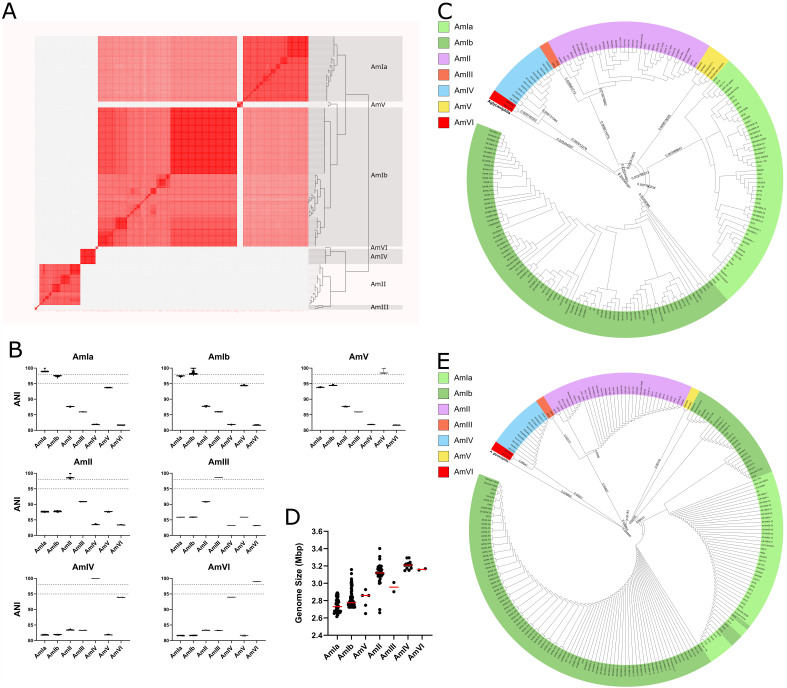
Phylogenetic analysis of *Akkermansia* genomes and evidence of speciation among seven major phylogroups. (A) ANI based on whole-genome comparisons, as calculated using pyANI in Anvi’o. The heatmap displays levels of ANI similarity between pairwise comparisons and grouping is set at a 95% similarity threshold. Red denotes a pairwise ANI of 95% or above. Major phylogroups are highlighted and labeled to maintain consistency with previous publications^[19-22]^. Note the presence of the new AmV and AmVI phylogroups; (B) Comparison of average ANI between *Akkermansia* phylogroups. Differences in average ANI below 95% were used to denote new species among phylogroups and an average ANI between 95% and 98% was defined to assign new sub-phylogroups within those new species (dotted lines on each subplot); (C) Phylogenetic analysis of core *Akkermansia* proteins. The predicted amino acid sequences encoded by single-copy *Akkermansia* genes were aligned and concatenated using anvi-get-sequences-for-gene-clusters. anvi-gen-phylogenomic-tree was used to generate a phylogenomic tree with FastTree. *A. glycaniphila* was used as an outgroup, and individual strains are highlighted by the proposed species and subspecies. Numbers denote main branch lengths; (D) Comparison of average (red line) genome sizes of high-quality complete genomes of representative members of *Akkermansia* phylogroups; (E) Phylogenetic grouping of full-length 16S rRNA *Akkermansia* sequences is consistent with genomic ANI comparisons. Full-length 16S rRNA gene sequences were extracted from genome sequences within Anvi’o. Clustal Omega was used to perform the alignment of those sequences, and the resulting phylogenetic tree was used for visualization. *A. glycaniphila* was used as an outgroup, and individual strains have been highlighted by phylogroup. ANI: Average nucleotide identity.

The average nucleotide identity (ANI) between two pairs of genomes has become the gold standard for identifying the genetic boundary of species from genome sequencing data^[61,62]^. Our pairwise analysis ANI between *Akkermansia* genomes reinforced the previously established phylogroup structure^[19,20,22]^ and added a new phylogroup [Figure 1A and Supplementary Table 2]. This phylogroup, which we termed AmVI, is represented by *Akkermansia* isolated from a patient undergoing cancer immunotherapy and a patient with amyotrophic lateral sclerosis. Current standards use 95% ANI as a threshold to define new species^[61,62]^, and a 98% ANI threshold has been used to define sub-species^[63]^. We calculated the average ANI between each pair of phylogroups and determined that the previously proposed phylogroups of *A. muciniphila* should be considered as separate species based on the 95% threshold, with *Akkermansia* AmI being considered *A. muciniphila* *sensu stricto*. At a 98% threshold, *A. muciniphila* splits into two sub-species: AmIa and AmIb [Figure 1B]. Two genomes that belonged to the AmIII group, BAA-2869 (isolated from squirrel) and CSUN-56 (isolated from human), each share an average ANI of less than 90% with all other phylogroups, indicating that these represent additional *Akkermansia* species [Supplementary Figure 1]. We have chosen to exclude the BAA-2869 and CSUN-56 genomes from further analyses, as they are likely single representatives of new species.

We next refined the evolutionary relatedness among *Akkermansia* genomes by identifying a total of 137 single-copy, core gene clusters among *Akkermansia* groups. We included *A. glycaniphila* to anchor into a deeper branch of the *Akkermansia* evolutionary tree. The aligned amino acid sequences predicted to be encoded by these gene clusters were concatenated to generate a phylogenetic tree. After rooting the tree to *A. glycaniphila*, we found a similar clustering of *Akkermansia* core proteins into the same phylogroups as predicted by ANI [Figure 1C].

As further evidence of the distinctiveness of *Akkermansia* species, an ANOVA test indicated that there are statistically significant differences in their average genome sizes [F(6,225) = 92.77, *P* < 0.0001]. *A. muciniphila* genomes were the smallest, with AmIa and AmIb displaying averages of 2.743 Mbp and 2.816 Mbp, respectively. Similarly, AmV had an average genome size of 2.809 Mbp. *A. massiliensis*, AmIII*,* and AmVI were larger with average genome sizes of 3.112, 2.956, and 3.161 Mbp, respectively [Figure 1D]. *A. biwaensis* had the largest genomes with an average size of 3.213 Mbp.

The divergence of 16S rRNA sequence identity is commonly used to distinguish between species^[64,65]^.We successfully extracted full-length representative 16S rRNA gene sequences for 217 genomes and generated a phylogenetic tree rooted on *A. glycaniphila*. Genomes for which our analysis did not yield full-length 16S rRNA gene sequences were of contig or scaffold assembly levels, suggesting incomplete assembly or low sequencing quality for these genomes. As with genome level ANI and core protein divergence, *Akkermansia* 16S rRNA sequences clustered by phylogroup, except for AmIa and AmIb sub-phylogroups, which could not be resolved. While these observations further support the phylogroup-level separation by ANI and core phylogeny, the average 16S rRNA gene sequence identity between any pair of phylogroups does not meet the commonly used threshold of 97%. At a higher species demarcating threshold of 98.65%, only *A. biwaensis* and the AmVI phylogroup together would be considered a new *Akkermansia* species [Figure 1E, Supplementary Figure 2 and Supplementary Table 3].

### *Akkermansia* species are differentiated by their core genomes and predicted metabolic function

**Figure 2:**
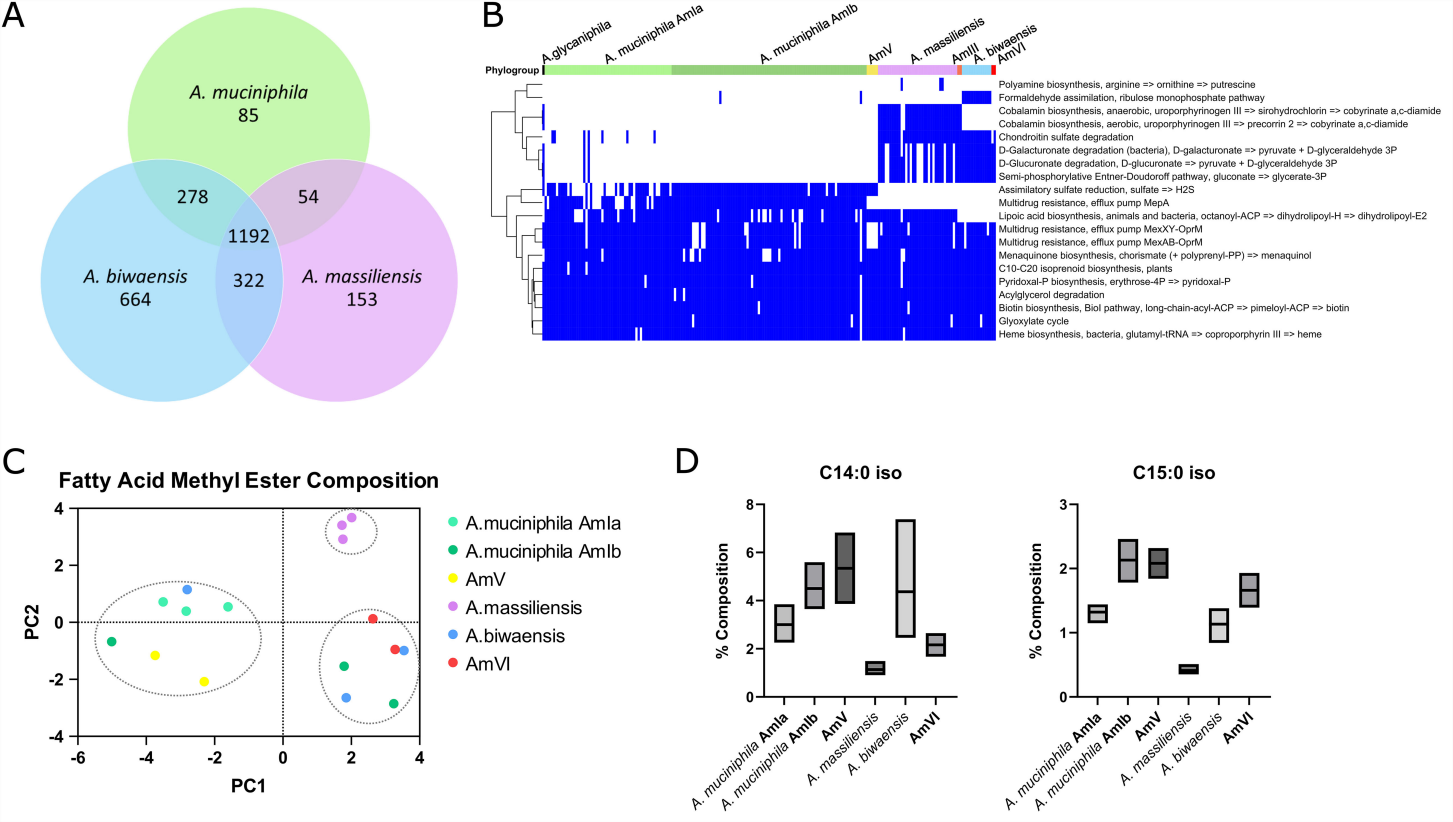
*Akkermansia* species are distinguishable by their estimated metabolic capabilities and fatty acid composition. (A) Unique core gene clusters were defined as those present in all genomes of one species, but not present in every genome of another species. Shared core gene clusters (*n* = 1,192) were defined as those present in all genomes of multiple species. All core gene clusters were categorized by these definitions and tallied to define the sizes of the *A. muciniphila*, *A. massiliensis*, and *A. biwaensis* core genomes; (B) Estimated metabolic enrichment analysis indicates the gain or loss of some metabolic pathways in *Akkermansia* phylogroups. Blue indicates pathway presence, calculated by pathway completeness of 50% or greater; (C) A principal components analysis was performed to compare fatty acid composition across species. The principal component scores distinguish three clusters comprised primarily of either *A. muciniphila* and AmV, *A. massiliensis*, or *A. biwaensis* and AmVI, as indicated by dotted ellipses. Isolates are colored by species; (D) Average fatty acid composition by C14:0 iso and C15:0 iso distinguishes *A. massiliensis* from other *Akkermansia* species.

To further differentiate between *A. muciniphila*, *A. massiliensis*, and *A. biwaensis*, we first identified gene clusters that were present in every genome of a single species, but not in the other species. We next identified gene clusters that were present in all genomes of two species, but not the remaining, and gene clusters present in all genomes of all three species [Figure 2A]. We find that 43% of these gene clusters (1,192/2,748) are present in all three species. *A. biwaensis* contains 664 gene clusters which are not core to *A. muciniphila* or *A. massiliensis*, *A. massiliensis* contains 153, and *A. muciniphila* only contains 85. That is, while 95% (1,524/1,609) and 91% (1,568/1,721) of the total *A. muciniphila* and *A. massiliensis* core genomes, respectively, are shared with the other species, only 73% (1,792/2,456) of the total *A. biwaensis* core genome is shared. This decrease in core genome size from *A. biwaensis* to *A. massiliensis* to *A. muciniphila* correlates with the relatedness of these species relative to *A. glycaniphila*.

To define the predicted metabolic capabilities of the new *Akkermansia* species and phylogroups, we assigned functions to predicted ORFs based on the KEGG Orthology (KO). The resulting KOs were filtered to include only those annotated as present or absent in at least three genomes in each phylogroup [Figure 2B]. As previously reported, gene clusters involved in assimilatory sulfate reduction are absent in *A. massiliensis* and *A. biwaensis*^[20]^, and gene clusters involved in cobalamin biosynthesis are unique to *A. massiliensis*^[22]^. Additionally, *A. biwaensis* is enriched with genes involved in formaldehyde assimilation but lacks lipoic acid biosynthetic genes. *A. massiliensis* and *A. biwaensis* are enriched with genes associated with chondroitin sulfate degradation, while multidrug resistance pumps present in the AmIa and AmIb phylogroups are missing from *A. massiliensis*, *A. biwaensis*, or AmV *Akkermansia*. The predicted metabolic capabilities of AmIII are largely the same as those of *A. massiliensis*, except that AmIII lacks genes for lipoic acid biosynthesis. Likewise, AmVI is similar to *A. biwaensis*; however, the AmVI strains are not enriched with genes related to formaldehyde assimilation.

### Phenotypic characterization of *Akkermansia* species

Analysis of *Akkermansia* pangenomes suggests there is significant diversity among *Akkermansia* species in terms of metabolic function^[20,22,66]^. Additional studies corroborated the phenotypic diversity between isolates of these species *in vitro*^[20,23]^. Two studies further characterized a few selected isolates using biochemical test kits and fatty acid methyl ester (FAME) analysis^[29,30]^. However, these studies were restricted to a single strain of *A. muciniphila* (Muc^T^), *A. glycaniphila* (Pyt^T^, isolated from python), and one isolate of either *A. massiliensis* or *A. biwaensis*. To account for strain differences within species, we performed FAME analysis and API 20A biochemical assays on 16 *Akkermansia* isolates, incorporating multiple isolates from each species and AmI phylogroups.

Fatty acid composition is a recommended means to phenotype microorganisms and has been used to differentiate closely related species, such as those of *Legionella*^[67,68]^. FAME analysis was performed on six representative *A. muciniphila* isolates (including Muc^T^, all isolated from humans), in addition to two AmV (isolated from mice), three *A. massiliensis* (isolated from humans), three *A. biwaensis* (isolated from humans), and two AmVI (isolated from humans) isolates. To compare the results across phylogroup, we then performed a Principal Component Analysis (PCA) [Figure 2C and Supplementary Table 4]. Three clusters of isolates were distinguished from PC1 and PC2, which account for 61.61% of the overall variance. These clusters primarily consist of (i) *A. muciniphila* and AmV, (ii) *A. massiliensis*, and (iii) *A. biwaensis* and AmVI. Of the fatty acids present at an abundance greater than 1%, we found that C14:0 iso and C15:0 iso distinguished *A. massiliensis* from the other species [Figure 2D and Supplementary Figure 3]. Distinguishing between the *A. muciniphila* and *A. biwaensis* by individual fatty acids was not possible.

Next, we used the API 20A system to perform biochemical phenotyping on six *A. muciniphila* (including Muc^T^, isolated from humans), two AmV (isolated from mice), three *A. massiliensis* (isolated from humans), four *A. biwaensis* (isolated from humans), and one AmVI (isolated from humans) isolate [Supplementary Table 5]. Out of the 21 assays performed, 15 yielded negative results for all isolates. All isolates were positive for glucose and lactose utilization, and the remaining four assays yielded varied results. Mannitol utilization was absent in all AmV, *A. massiliensis*, and AmVI isolates, but varied between isolates of *A. muciniphila* and *A. biwaensis*. Maltose utilization occurred in both AmV isolates, not in the AmVI isolate, and varied between isolates of *A. muciniphila*, *A. massiliensis*, and *A. biwaensis*. All isolates of AmV and *A. biwaensis* could use mannose but not the AmVI isolate, and mannose utilization varied between isolates of *A. muciniphila* and *A. massiliensis*. Catalase activity was present in all isolates of AmV and *A. massiliensis*, absent in the AmVI isolate, and varied between isolates of *A. muciniphila* and *A. biwaensis*. Interestingly, this variability in catalase activity correlates to the variable, low, and high sensitivity to oxygen demonstrated by *A. muciniphila*, *A. massiliensis*, and *A. biwaensis* in a prior study^[20]^.

### *Akkermansia* species and subspecies-level assignments can be made from metagenomic sequences and 16S rRNA V3-V4 regions

*Akkermansia* species and phylogroups have distinct metabolic, *in vitro* phenotypic, and *in vivo* competitive characteristics^[20-23]^ that could be linked to health or disease risks. We next asked if we could use existing 16S rRNA and metagenomic sequencing data generated from various patient cohorts to clarify the relationships between *Akkermansia* species and human health. We leveraged our pangenomic analysis results to enhance existing metagenomic sequencing tools, enabling us to achieve species-level resolution of the *Akkermansia* in these published datasets.

To determine if existing strain-finding programs can distinguish between *Akkermansia* species, we used the POMMS stool samples^[20,40]^, for which we have matching 16S rRNA amplicon and shotgun metagenomic sequencing datasets. We also previously isolated *Akkermansia* strains from several of these samples^[20]^. We identified *Akkermansia* phylogroups from metagenomic sequencing data using a combination of three strain-finding programs, StrainPhlAn3, StrainR, and SMEG^[53,55,56]^.

StrainPhlAn3 is broadly used to identify bacterial strains from metagenomic data^[69]^. To identify *Akkermansia* phylogroups, we ran metagenomic sequencing data generated from POMMS samples through our StrainPhlAn3 workflow, which included 31 samples from which we had previously identified and isolated the dominant *Akkermansia* species^[20,70]^. StrainPhlAn3 frequently failed to detect *Akkermansia* in samples where the relative abundance was less than 0.5% of total bacterial sequences and was able to assign a phylogroup to only 85 out of 146 POMMS metagenomes with levels of *Akkermansia* detectable by MetaPhlAn3 [Supplementary Figure 4A].

**Figure 3:**
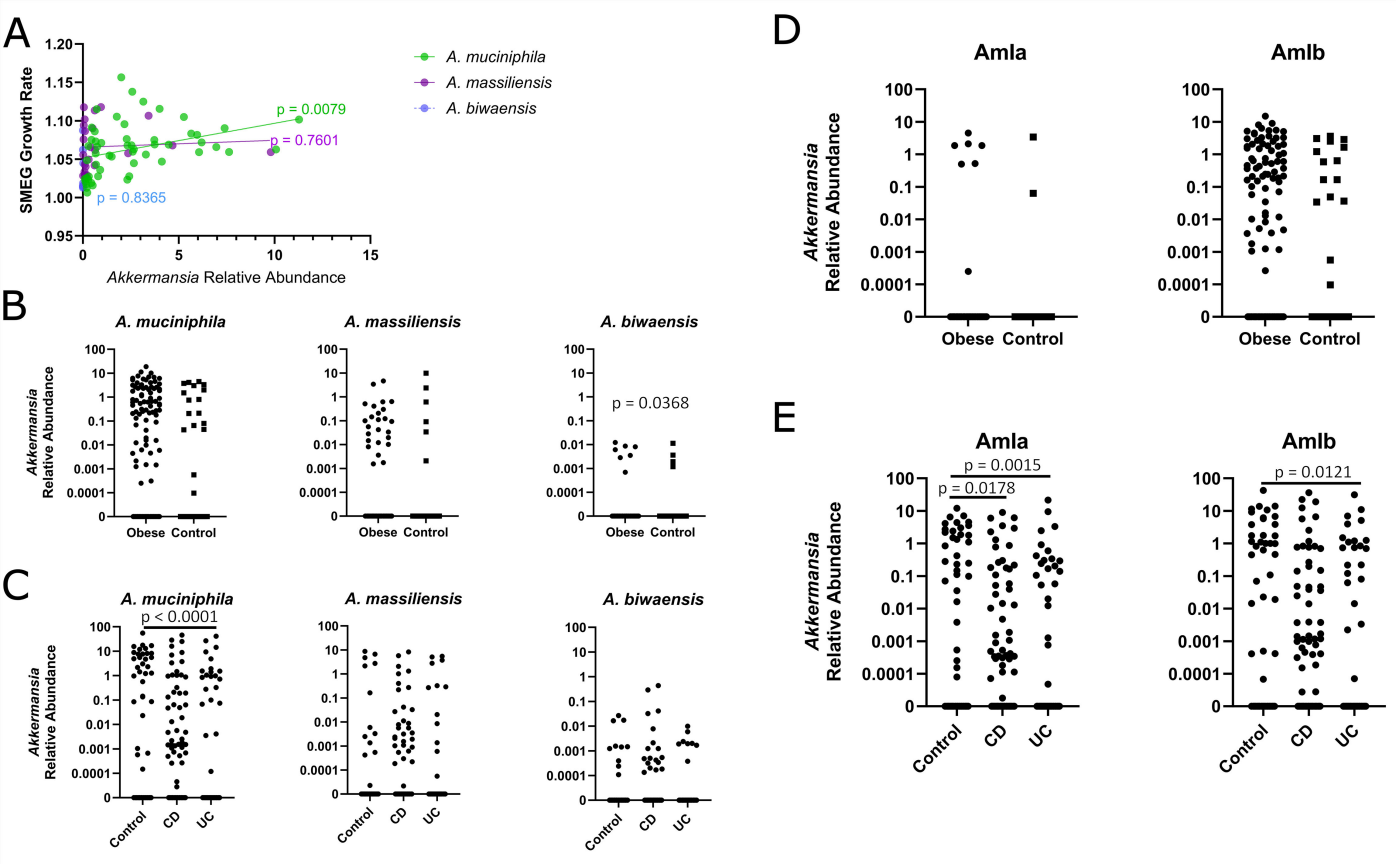
The relative abundance of the three main *Akkermansia* species and *A. muciniphila* sub-phylogroup in two different patient cohorts can correlate with disease outcomes. (A) *In vivo* growth rate is a minor contributor to the relative abundance of *Akkermansia* species in stool samples. A simple linear regression was performed to determine if the relative abundance of *Akkermansia* in a metagenomics sequencing sample is predictive of the estimated growth rate as determined by SMEG. Each regression line is colored by, and represents, either *A. muciniphila*, *A. massiliensis*, or *A. biwaensis*; (B) Comparison of the relative abundances of *A. muciniphila*, *A. massiliensis*, and *A. biwaensis* between children with obesity, defined as having a gender-specific BMI greater than the 95th percentile, and control samples from the POMMS dataset; (C) Comparison of the relative abundances of *A. muciniphila*, *A. massiliensis*, and *A. biwaensis* between healthy controls, CD, and UC samples from the combined IBD dataset; (D) Comparison of the relative abundances of AmIa and *AmIb A. muciniphila* between children with obesity and control samples from the POMMS dataset; (E) Comparison of the relative abundances of AmIa and AmIb *A. muciniphila* between healthy controls, CD, and UC samples from the combined IBD dataset. SMEG: Strain-level metagenomic estimation of growth rate; BMI: body mass index; POMMS: Pediatric Obesity Microbiome and Metabolism Study; UC: ulcerative colitis; CD: Crohn’s disease.

SMEG is designed to identify bacterial strains and measure their growth rate based on metagenomic sequences by calculating the coverage of SNPs closer to the origin of replication as compared to those closer to the terminus region^[56]^. We generated a SMEG database using 34 *Akkermansia* genomes representing different species and strains and used the growth_est module to predict *Akkermansia* species. SMEG was able to assign a species or phylogroup to 108 out of 146 POMMS metagenomic samples with detectable (greater than 0% relative abundance) levels of *Akkermansia*, which allowed for improved sensitivity in assigning phylogroups and for the estimation of growth rates *in vivo*. The results of a simple linear regression indicate that the relative abundance of *A. muciniphila* is a significant predictor of its estimated growth rate [R^2^ = 0.1281, F(1,52) = 7.642, *P* = 0.0079] [Figure 3A]. However, no significant association between growth rate and relative abundance was found for either *A. massiliensis* [R^2^ = 0.0043, F(1,22) = 0.0956, *P* = 0.7601] or *A. biwaensis* [R^2^ = 0.0094, F(1,5) = 0.0473, *P* = 0.8365]. Several samples display high growth rates despite having a low abundance of *A. massiliensis* or *A. biwaensis*. Additionally, the relative abundance of *A. muciniphila* explains only 12.81% of the variation in the estimated growth rate. Thus, other factors, such as predation by phages, interbacterial competition, or differential adherence to the GI epithelia, may play significant roles in determining the prevalence of *Akkermansia* species in stool.

StrainR is designed for the relative quantification of highly related strains from metagenomic datasets^[55]^. We generated a StrainR database using one representative *Akkermansia* genome per species and phylogroup, and StrainR assigned a species or phylogroup to 139 out of 146 POMMS metagenomic samples with detectable levels of *Akkermansia*, nearly doubling the sensitivity of StrainPhlAn3. Out of those 146 POMMS samples with detectable levels of *Akkermansia*, 138 (94.52%) were found to contain a single *Akkermansia* species. The remaining eight samples all contained *A. muciniphila* in addition to *A. massiliensis* and/or *A. biwaensis*. Of the samples containing only *A. muciniphila*, 92 were predicted to contain both AmIa and AmIb*.* The ratio of AmIa and AmIb was consistent (~23%/72%) and independent of the total relative abundance of *A. muciniphila* in the samples [Supplementary Figure 4B]. This could either mean that StrainR is unable to reliably distinguish between AmIa and AmIb or the less likely scenario that AmIa and AmIb strains co-occur at fixed ratios.

To validate the effectiveness of these methods, the *Akkermansia* species and subspecies in POMMS patients were compared against strains isolated from the corresponding stool samples. Out of 31 POMMS metagenomic samples with matching isolates, we identified 26 samples with measurable levels of *Akkermansia*, as assessed by MetaPhlAn3, for which we could predict the prevalent phylogroup by a combination of StrainPhlAn3, StrainR, and SMEG. For these samples, 24/26 (92%) of the isolated strains matched the predicted phylogroup. Eight samples in the entire dataset were predicted to contain more than one *Akkermansia* species, accounting for a range of 1.57%-33.50% and a median of 13.83% of *Akkermansia* abundance of the additional minor species. Note that this method has only been validated to distinguish between the *A. muciniphila*, *A. massiliensis*, and *A. biwaensis* species, as the validation dataset did not include *Akkermansia* AmIII, AmV, or AmVI isolates.

We noted that full-length 16S rRNA gene sequences clustered by the new proposed *Akkermansia* species nomenclature. We determined that this clustering is maintained if we restrict our analysis to the V3-V4 subregion, which is commonly used in 16S rRNA gene-based microbial community profiling [Supplementary Figure 5]. Therefore, we looked at 16S rRNA V3-V4 sequences generated in the POMMS study to determine if we can make species-level assignments in this cohort. The sequences of amplified sequence variants (ASVs) annotated as genus *Akkermansia* were extracted and aligned to 16S rRNA gene sequences from our reference strains, then assigned to new *Akkermansia* species, depending on with which isolate their sequences clustered. We then compared the predicted phylogroups to the known species in those same samples. Out of 34 samples with both 16S rRNA sequencing data and an isolated strain, the predicted and isolated species matched in 10 samples. In 21 samples, an additional phylogroup was identified. While there may be more than one *Akkermansia* strain present in the original stool, we mostly isolated one strain from any one sample^[20]^ and the predicted phylogroups from 16S rRNA gene sequences matched in 31/34 (91%) of cases. Overall, our validation of both 16S rRNA and metagenomic analysis suggests a high accuracy in identifying the most prevalent human *Akkermansia* species.

### Correlations between *Akkermansia* species and health in metagenomic studies

We reanalyzed metagenomic datasets of patients with obesity, inflammatory bowel disease, and responsiveness to cancer immunotherapies - instances where *Akkermansia* has been reported to influence health - to determine if there are correlations between specific species of *Akkermansia* and the health status of their host. Among all the samples analyzed where *Akkermansia* sequences were detectable, we found *A. muciniphila* to be the most prevalent species (354/1,088, 32.54%), followed by *A. massiliensis* (11.86%) and *A. biwaensis* (5.15%). In some of those samples (78/1,088, 7.17%), more than one *Akkermansia* was present. Most samples (648/1,088, 59.56%) did not have measurable levels of *Akkermansia*.

There is a negative correlation between *Akkermansia* abundance and obesity in adult humans and mouse models of diet-induced obesity^[3-5,7,71]^. We performed Mann-Whitney U tests to compare the relative abundances of *A. muciniphila*, *A. massiliensis*, or *A. biwaensis* between children with obesity (case) and control samples from this same dataset [Figure 3B]. There was no significant difference in the relative abundance of *A. muciniphila* (*U* = 6,971, *P* = 0.1569) or *A. massiliensis* between cases and controls (*U* = 7,367, *P* = 0.3087). There was a significant difference in *A. biwaensis* between case and control samples (*U* = 7,262, *P* = 0.0368); however, the low prevalence of *A. biwaensis* (8.16% of control samples and only 2.22% of case samples had detectable levels of *A. biwaensis*) limits the power of such analysis.

*Akkermansia* abundance has been linked to varying risk for IBD. *Akkermansia* has been found to be depleted in adult human cases of Crohn’s disease (CD) and ulcerative colitis (UC), while other studies have found *Akkermansia* to only be depleted in the case of CD (in pediatric patients), or to be associated with responsiveness to treatment rather than with disease occurrence^[31,72-74]^. Other multi-omics studies have found no significant association between *Akkermansia* and IBD^[43,44]^. We combined three of the largest adult-IBD metagenomic studies^[42-44]^ and processed them to make *Akkermansia* species and subspecies assignments. In this combined dataset, 34.64% of samples (124/358) contained detectable levels of *Akkermansia*.

Kruskal-Wallis H tests were performed to compare the relative abundances of *A. muciniphila*, *A. massiliensis*, or *A. biwaensis* between CD, UC, and control samples [Figure 3C], indicating that there is a statistically significant difference in the relative abundance of *A. muciniphila* between disease groups [H(3) = 20.08, *P* < 0.0001]. Pairwise comparisons using Dunn’s multiple comparisons test indicated that *A. muciniphila* is significantly decreased in the UC group relative to healthy controls (control mean rank = 207.1, UC mean rank = 157.8, *P* < 0.0001). Similarly, there is a significant difference in *A. massiliensis* relative abundance between groups [H(3) = 10.30, *P* = 0.0058]. Pairwise comparisons did not yield any statistically significant results, possibly due to the low prevalence of *A. massiliensis* in all groups (16.46% in controls, 8.66% in CD, and 8.33% in UC). We also found no significant difference in *A. biwaensis* relative abundance between groups [H(3) = 5.075, *P* = 0.0791].

We next asked if the relative abundance of *A. muciniphila* subspecies (AmIa and AmIb), influenced their correlation with health. In the POMMS dataset, there was no significant difference in the relative abundance of *A. muciniphila* AmIa (*U* = 7,574, *P* = 0.5084) or AmIb (*U* = 7,115, *P* = 0.2384) between case (obese) and control samples [Figure 3D]. For the combined IBD dataset [Figure 3E], we found that there is a significant difference in both AmIa [H(3) = 11.75, *P* = 0.0028] and AmIb [H(3) = 11.08, *P* = 0.0039] between healthy and IBD groups. Post hoc pairwise comparisons indicated that AmIa is significantly decreased in both the CD and UC groups (control mean rank = 192.8, CD mean rank = 177.9, *P* = 0.0178, UC mean rank = 173.8, *P* = 0.0015), while AmIb is only significantly decreased in the UC group relative to healthy controls (control mean rank = 192.4, UC mean rank = 163.3, *P* = 0.0121).

**Figure 4:**
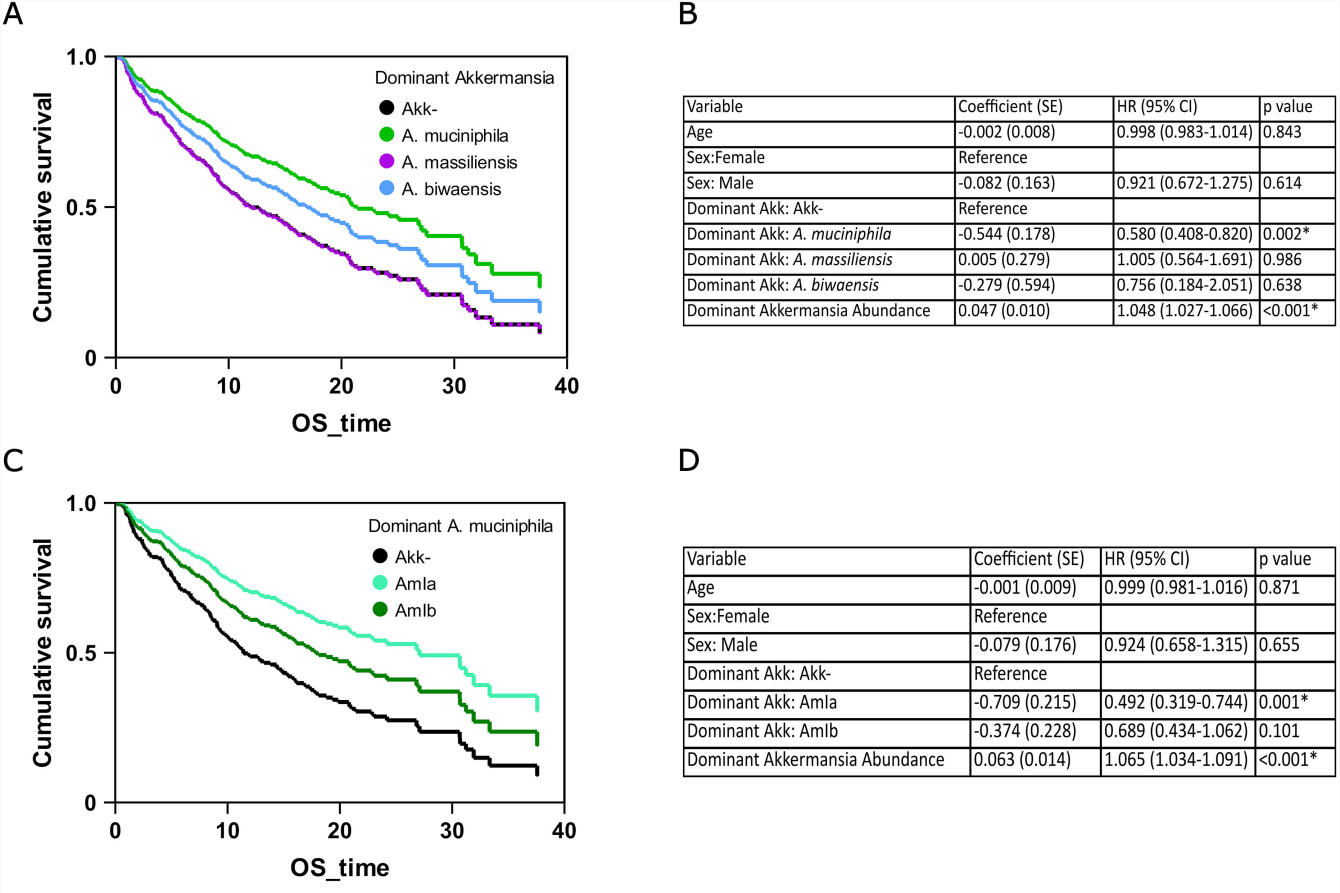
The association between *Akkermansia* and improved survival following PD-1 blockade treatment depends on the dominant *Akkermansia* species and sub-phylogroup. (A) A Cox regression model was used to determine the factors associated with prolonged survival after treatment for non-small-cell lung cancer. Cumulative survival is displayed using dominant *Akkermansia* species as the predictive variable, colored by species. The line for *A. massiliensis* is dotted to make the line for *Akkermansia*-negative samples visible; (B) The tabular results of the Cox regression model reveal that the relationship between *Akkermansia* and prolonged survival is specific to *A. muciniphila* (AmI); (C) A Cox regression model was used to determine the factors associated with prolonged survival after treatment for non-small-cell lung cancer. Cumulative survival is displayed using the dominant *A. muciniphila* phylogroup as the predictive variable; (D) The tabular results of the Cox regression model reveal that the relationship between *A. muciniphila* and prolonged survival is specific to the AmIa phylogroup.

*Akkermansia* is associated with improved outcomes following PD-1 blockade treatment of epithelial tumors^[6]^ and the relative abundance of *Akkermansia* is predictive of clinical responsiveness to similar treatment of non-small-cell lung cancer (NSCLC)^[41]^. In particular, the presence of *Akkermansia* in patient stools was associated with improved responses to immunotherapy and overall survival, compared to patients with no *Akkermansia*. We reanalyzed stool metagenomes from one of these studies and assigned a dominant *Akkermansia* species and subspecies to each sample^[41]^. Based on a Cox regression model, adjusted for sex and age of patients [Figures 4A and B], we found a significant survival difference between patients colonized predominantly by *A. muciniphila* (AmI) and those without *Akkermansia* (HR = 0.580, 95%CI: 0.408-0.820, *P* = 0.002). There was no significant survival difference between patients colonized predominantly by *A. massiliensis* or *A. biwaensis*.

We also found a significant difference between *A. muciniphila* AmIa and AmIb in the survival of patients with NSCLC undergoing immune checkpoint inhibitor treatment. After restricting our analysis to those samples with detectable *A. muciniphila*, we generated a Cox regression model [Figures 4C and D] and found that patients colonized predominantly by AmIa had a significant increase in survival compared to patients with no detectable *A. muciniphila* (HR = 0.492, 95%CI: 0.319-0.744, *P* = 0.001). We did not detect this enhanced survival in patients predominantly colonized with *A. muciniphila* AmIb (HR = 0.689, 95%CI: 0.434-1.062, *P* = 0.101).

Overall, our findings indicate that in circumstances where *A. muciniphila* is associated with an impact on host physiology or immunity, which subspecies predominates can make a difference in its association with disease outcomes. In IBD, AmIa abundance is linked to protection from both CD and UC, while AmIb was only associated with protection from UC. Likewise, the increased survival noted in NSCLC patients is only significant for those patients who were colonized by predominantly AmIa.

## DISCUSSION

We performed a detailed analysis of genomic variance among human *Akkermansia* isolates and reinforced the existence of at least six phylogroups (AmI-AmVI)^[19-22]^. We also provide further support for the assignments of new *Akkermansia* species, *A. massiliensis* and *A. biwaensis*, and for at least two *A. muciniphila* sub-species groups. These conclusions are based on meeting the threshold of separate species as assessed by a 95% genome ANI threshold, which is further supported when comparisons are limited to proteins encoded by *Akkermansia* core genes. Species assignments have traditionally used a 97% threshold of 16S rRNA gene identity, and at a less restrictive threshold of 98.65%, only one new species would be formed, containing the AmIV and AmVI phylogroups. However, discrepancies between ANI and 16S rRNA gene identities when making species classifications are not uncommon. Genome-based classifications of *Burkholderia* and *Anaplasma* isolates have identified strains that rise to the level of new species by ANI, but not 16S rRNA genes, suggesting that the utility of 16S rRNA identity as a sole determinant of species classification is limited^[75,76]^. These studies suggest that whole genome data methodologies, such as producing single-copy core gene phylogenies and ANI clustering, are more appropriate for demarcating closely related species. Indeed, the AmII and AmIV phylogroups were recently renamed *A. massiliensis* and *A. biwaensis*, respectively^[23,29,30]^. The report that renamed *Akkermansia* AmII as *A. massiliensis* suggested that the AmIV phylogroup also met the criteria of a new species and proposed the name “*Candidatus* Akkermansia timonensis”. However, several AmIV strains had been previously isolated and their genomes sequenced, making the *Candidatus* term inappropriate^[20,60]^. Overall, the results of our study indicate that *A. muciniphila* should be split into several species including *A. muciniphila*, *A. massiliensis*, and *A. biwaensis*. The AmV, AmIII, and AmVI groups should be defined as novel species as well [Table 1]. We propose that the AmIa phylogroup of *A. muciniphila* be renamed as *A. muciniphila* subsp. *muciniphila*, given that the type strain of *A. muciniphila*, Muc^T^, belongs to this subspecies. We propose that the AmIb phylogroup be renamed as *A. muciniphila* subsp. *communis* (kom’mu.nis. L. m. adj. *communis* common, referring to this subspecies being more prevalent than subsp. *muciniphila* among MAGs and isolates). The type strain subsp. *communis* Akk1570^T^ was isolated from the stool of a child with obesity. Given that two AmV isolates were from mice with different chronic intestinal inflammatory disorders^[21]^, we propose that this phylogroup be renamed *Akkermansia ignis* (ig.nis. L. m. n. *ignis* fire, referring to inflammation). The type strain MmAkk2^T^ was isolated from mouse stool*.* Similarly, since both AmVI isolates were derived from patients in Durham NC, we propose to rename this phylogroup as *Akkermansia durhamii* (dur.ham’i.i, N.L. m. n. *durhamii*, referring to the city of Durham where these strains were isolated). The type strain is RCC_12PD^T^ and was isolated from the stool of a patient with RCC. Both genomic and FAME analysis of multiple isolates indicate that *A. ignis* is more closely related to *A. muciniphila*, and *A. durhamii* is more related to *A. biwaensis*. Though reclassifying the AmIII group as a new species is appropriate based on the described genomic analysis, our laboratory does not have access to either of the only two isolates described thus far (GP22 and GP24). Because there is currently no type strain available for public use and our laboratory has not isolated an AmIII strain of our own, we were unable to perform further phenotypic characterization of this group. Thus, we have chosen not to suggest a new species epithet or type strain.

**Table 1:** Proposed nomenclature for *Akkermansia* phylogroups

**Previous nomenclature**	**Proposed nomenclature**	**Type strain**	**Type strain availability**	**Average genome size (Mbp)**
*A. muciniphila* AmIa	*A. muciniphila* subspecies *muciniphila*	Muc^T^	ATCC BAA-835 = DSM 22959	2.743
*A. muciniphila* AmIb	*A. muciniphila* subspecies *communis*	Akk1570^T^		2.816
*A. muciniphila* AmII	*A. massiliensis*	Marseille-P6666^T^	CSUR P6666 = CECT 30548	3.112
*A. muciniphila* AmIII				2.956
*A. muciniphila* AmIV	*A. biwaensis*	WON2089^T^	NBRC 115679 = DSM 114407	3.213
*A. muciniphila* AmV	*A. ignis*	MmAkk2^T^	*Submitted to ATCC and DSMZ*	2.809
*A. muciniphila* AmVI	*A. durhamii*	RCC_12PD^T^	*Submitted to ATCC and DSMZ*	3.161

Our analysis of *Akkermansia* genomes suggests that the seven major phylogroups, all previsouly classified as *A. muciniphila*, represent six species in total. The nomenclature proposed in this and past publications, availability of proposed species type strains, and average proposed species genome size are listed for each of the seven major clades.

We noted marked differences in the genome sizes of the genus *Akkermansia*, with *A. massiliensis*, *A. biwaensis*, and *A. durhamii* displaying genomes > 15% larger than *A. muciniphila*. Based on our phylogenetic analysis, it appears that the *A. biwaensis/A. durhamii* split from the *A. massiliensis*/*A. muciniphila* branches, and that these branches evolved separately, with the smaller *A. muciniphila* becoming most prominent in human samples worldwide [Figure 1A]. The relative abundance of *A. muciniphila* strains is also higher in human stool samples than other *Akkermansia* species, although their growth rates, as assessed by SMEG, are relatively equivalent [Figure 3A]. This suggests that factors unrelated to replication rates (e.g., phage predation, susceptibility to host and microbiota antimicrobial compounds) may be a more prominent driver of overall *Akkermansia* abundance in the GI tract.

While different methodologies have been applied for the pangenomic analysis of the *Akkermansia* genus to define relationships between *Akkermansia* and health, these have not generally taken into consideration subspecies and strain assignments^[22,25,27,66,77]^. This could be particularly relevant when considering the potential of *Akkermansia* strains to be developed into next-generation probiotics^[13-16]^. The new species outlined, both as previously suggested^[20,22,23,60]^ and reinforced by the results presented in this study, highlight that there are clear phenotypic differences that may be relevant to the interaction between *Akkermansia* and the host. Indeed, we find species and subspecies level assignments refine the outcome of associations between *Akkermansia* and humans, where one sub-species may drive the associations observed in the past, as in the case of increased responsiveness to PD-1 blockade in patients colonized by AmIa *Akkermansia*. Thus, targeting specific strains to be developed into future probiotics should include an analysis of which phylogroup is most strongly associated with the health benefit of interest. Additionally, if one or more phylogroups of *Akkermansia* are determined to be negatively associated with health outcomes in a particular context, knowing what strains to avoid during therapeutic development will decrease the risk of failure or development of adverse effects.

Our study presents certain limitations. First, the novel species described in our analysis of the *Akkermansia* pangenome are relatively rare in the human populations we have characterized and appear to be present in lower abundance than other *Akkermansia* species. There are currently only five isolates of *A. ignis* and two of *A. durhamii*. Two strains, BAA-2860 and CSUN-56, may also represent novel species. Prescreening of MAGs generated from metagenomic sequencing of fresh stool samples would allow for more efficient, targeted isolation of these rare species. The functional characterization of *Akkermansia* strain by standard methods like the API 20A system is of limited use as the composition of the assay medium is not compatible with culturing *Akkermansia*. Indeed, the species identification table provided by bioMérieux does not include *Akkermansia*. There are also some limitations to the use of StrainR in reliably distinguishing between the *A. muciniphila* AmIa and AmIb subspecies.

Finally, the rarity of the non-*A. muciniphila* species can decrease the statistical power of any associations between these species and human health conditions. As illustrated in cases of pediatric obesity, the proportion of individuals with a detectable level of *A. biwaensis* was low, which, compounded with the low prevalence of these new species compared to *A. muciniphila*, makes determining relevant disease associations more challenging. Thus, much larger datasets will be necessary to properly investigate the importance of these new *Akkermansia* species to human health.

## DECLARATIONS

### Availability of data and materials

Genomes retrieved from NCBI and their accession numbers are listed in Supplementary Table 1. 16S and metagenomic datasets for method validation were provided by the POMMS^[40]^. Metagenomic sequencing samples case studies were obtained from the SRA using Bioproject accessions PRJNA398089, PRJNA400072, PRJEB42151, PRJEB42155, PRJNA751792, and PRJNA782662^[41-44]^. Sequences for the *Akkermansia durhamii* isolates have been deposited in GenBank under BioProject accession number PRJNA1066260.

### Copyright

© The Author(s) 2024.
