# Supplementary material for: Pangenomic analysis identifies correlations between Akkermansia species and subspecies and human health outcomes
Source: Microbiome Res Rep. 2024 Jun 11;3(3):33. doi: 10.20517/mrr.2024.09 (PMC11480726; doi:10.20517/mrr.2024.09)
Supplement: Supplementary file 1 [file mrr-3-3-33-SupplementaryMaterials.zip › mrr3009-SupplementaryMaterials/Supplementary Figures.pdf]

Mueller *et al. Microbiome Res Rep* 2024;3:33
DOI: 10.20517/mrr.2024.09

Microbiome Research Reports

Supplementary Figures

Pangenomic analysis identifies correlations between *Akkermansia* species and subspecies and human health outcomes

Katherine D. Mueller[1,2], M. Emilia Panzetta[1,2], Lauren Davey[3], Jessica R. McCann[4], John F. Rawls[2,4], Gilberto E. Flores[5], Raphael H. Valdivia[1,2]

[1]Department of Integrative Immunobiology, Duke University, Durham, NC 27710, USA.

[2]Duke Microbiome Center, Duke University School of Medicine, Durham, NC 27710, USA.

[3]Department of Biochemistry and Microbiology, University of Victoria, Victoria V8P 5C2, British Columbia, Canada.

[4]Department of Molecular Genetics and Microbiology, Duke University, Durham, NC 27710, USA.

[5]Department of Biology, California State University, Northridge, CA 91330, USA.

Correspondence to: Dr. Raphael H. Valdivia, Department of Integrative Immunobiology, Duke University, 3110 MSRB3, 3 Genome Court, Durham, NC 27710, USA. E-mail: raphael.valdivia@duke.edu

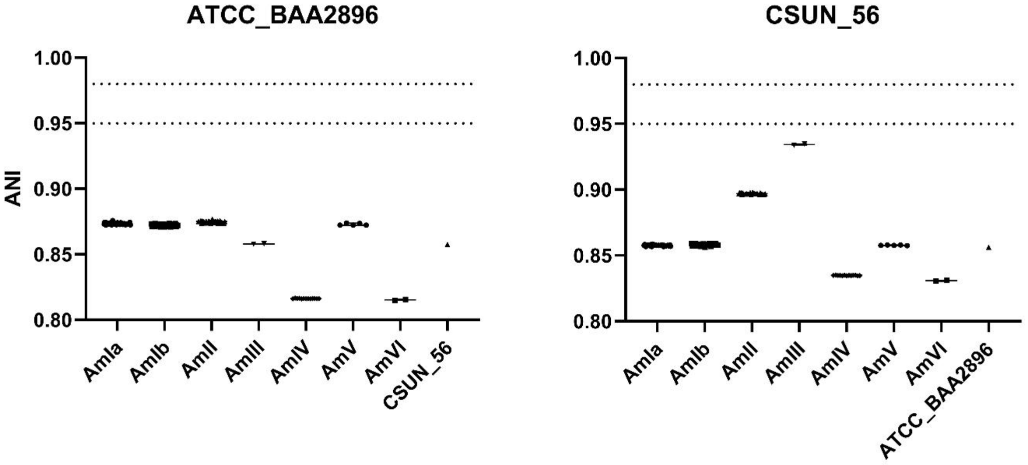

Supplementary Figure 1. Analysis of average nucleotide identity suggests that two strains previously classified as AmIII may represent two novel species. Average nucleotide identity (ANI) based on whole-genome comparisons between ATCC BAA2896, CSUN-56, and all other *Akkermansia* phylogroups, as calculated using pyANI in Anvi'o. Differences in average ANI below 95% were used to denote new species among phylogroups, and an average ANI between 95% and 98% was defined to assign new sub-phylogroups within those new species (dotted lines on each subplot). From this, it may be determined that ATCC BAA2896 and CSUN-56 should each be considered single representatives of new species.

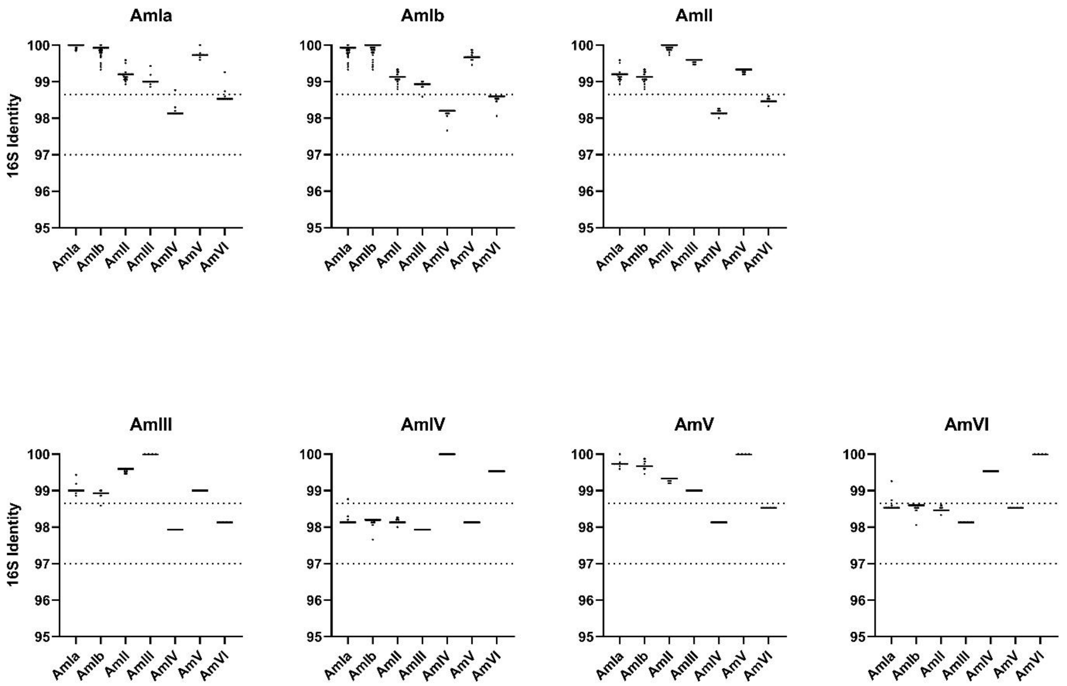

Supplementary Figure 2. The average 16S rRNA gene sequence identity between phylogroups does not meet commonly used thresholds for delineating species. Full-length 16S rRNA gene sequences were extracted from genome sequences within Anvi'o. Clustal Omega was then used to compare the average sequence identity between phylogroups. The dotted lines indicate 97% and 98.65% identity, which are commonly used thresholds to delineate species.

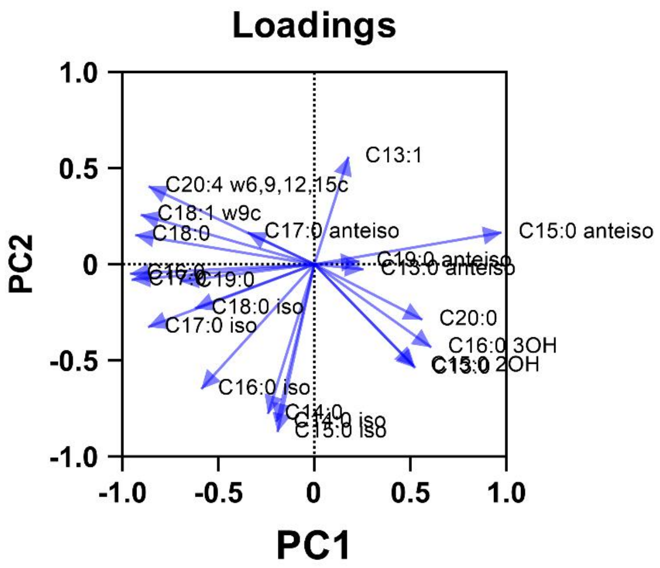

Supplementary Figure 3. Loadings from a principal components analysis suggest that the factors driving separation across PC2 include C14:0 iso and C15:0 iso. A principal components analysis was performed to compare fatty acid composition across species. The biplot [Figure 2A] revealed that *A. massiliensis* could be clearly separated from other *Akkermansia* species along PC2. Loadings indicate that the most informative fatty acid features along PC2 include C14:0 iso and C15:0 iso.

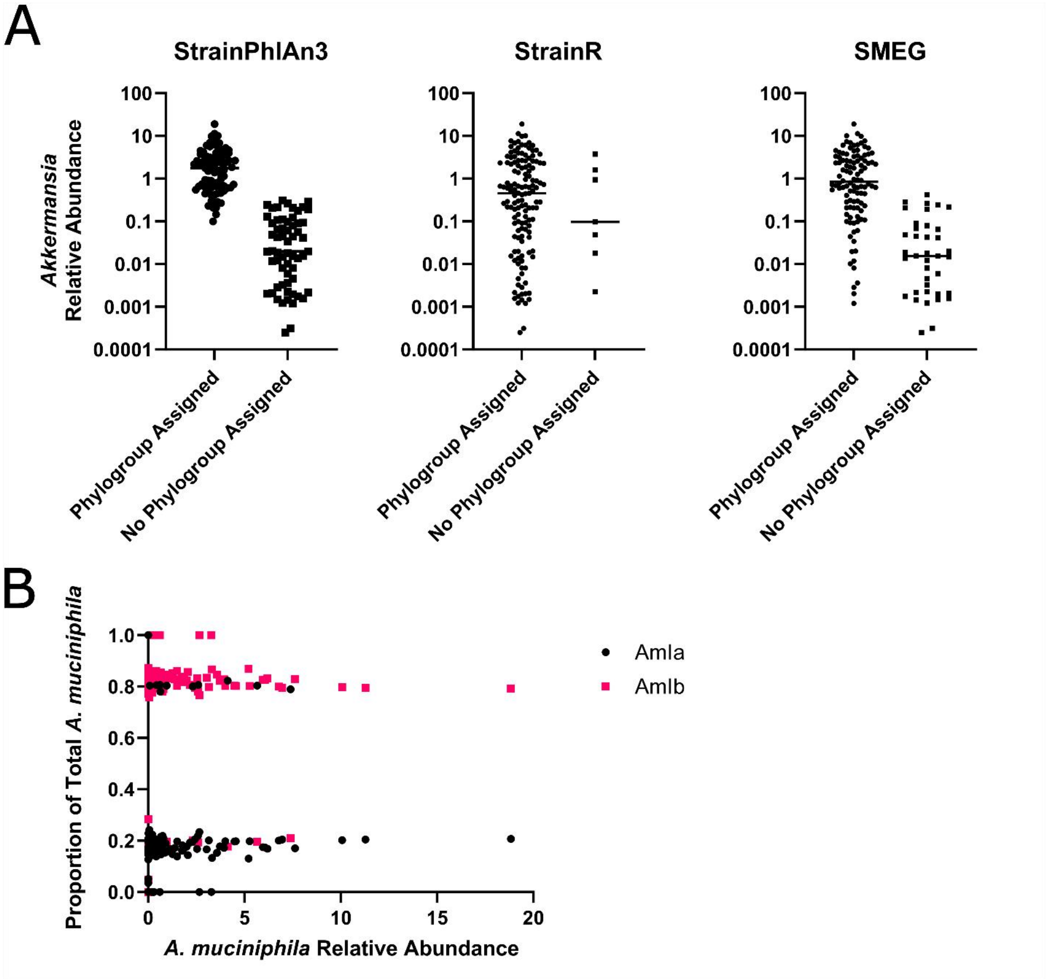

Supplementary Figure 4. Each of the strain-finding programs used in the identification of *Akkermansia* species from metagenomic sequencing data is imperfect. (A) StrainPhlAn3, StrainR, and SMEG were each run on the entire POMMS dataset to determine how many samples for an *Akkermansia* species could be identified. StrainPhlAn3 frequently did not detect *Akkermansia* in samples where the relative abundance was less than 0.5% of total bacterial sequences. StrainR and SMEG each identified *Akkermansia* species and phylogroups in the same low-abundance samples with greater rates of success; **(B)** The proportion of AmIa and AmIb present in samples, as calculated using StrainR, was determined according to the total relative abundance of AmI in metagenomic samples. Black indicates AmIa and pink indicates AmIb. Note that the proportion of these AmI phylogroups does not change in association with relative abundance.

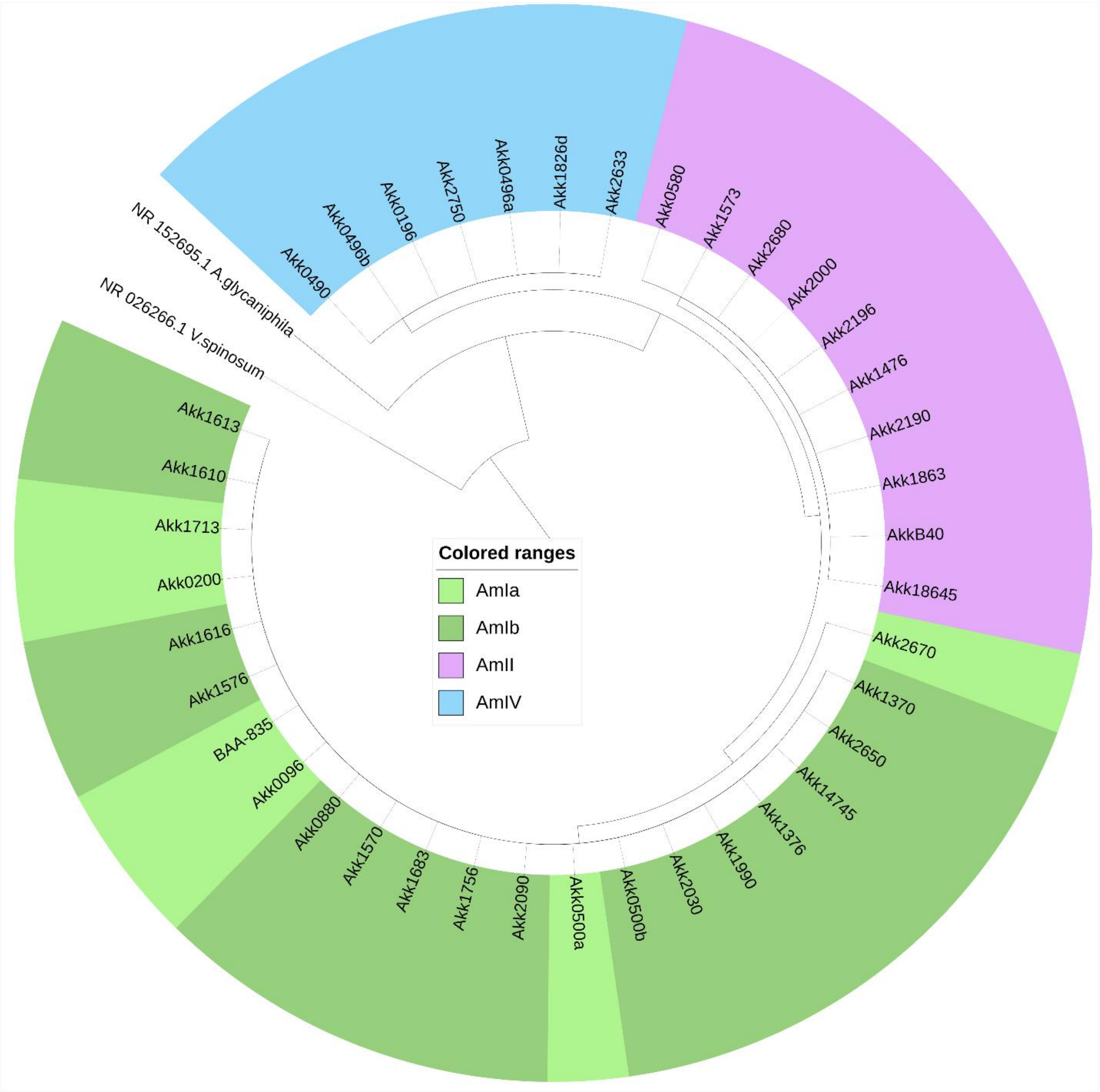

Supplementary Figure 5. Sub-phylogroups of *A. muciniphila* are not resolvable by their V3-V4 16S rRNA sequences. The V3-V4 region of the 16S rRNA gene sequences of 39 *Akkermansia* isolates were aligned using Clustal Omega, and the resulting phylogenetic tree was used for visualization. *A. glycaniphila* and *V. spinosum* were used as outgroups. Individual strains have been highlighted by species, showing clear clustering of *A. massiliensis* and *A. biwaensis*. However, the AmIa and AmIb isolates of *A. muciniphila* are not able to be differentiated by the V3-V4 region alone.
